# Identification of suitable reference genes for hepatic microRNA quantitation

**DOI:** 10.1186/1756-0500-7-129

**Published:** 2014-03-07

**Authors:** Vishal Lamba, Yogita Ghodke-Puranik, Weihua Guan, Jatinder K Lamba

**Affiliations:** 1 Department of Experimental and Clinical Pharmacology, University of Minnesota, Minneapolis, MN, USA; 2 Division of Biostatistics, School of Public Health, University of Minnesota, Minneapolis, MN, USA

**Keywords:** microRNAs, Hepatic, miRNA expression, Real-time quantification, Normalization, Endogenous controls

## Abstract

**Background:**

MicroRNAs (miRNAs) are short (~22 nt) endogenous RNAs that play important roles in regulating expression of a wide variety of genes involved in different cellular processes. Alterations in microRNA expression patterns have been associated with a number of human diseases. Accurate quantitation of microRNA levels is important for their use as biomarkers and in determining their functions. Real time PCR is the gold standard and the most frequently used technique for miRNA quantitation. Real time PCR data analysis includes normalizing the amplification data to suitable endogenous control/s to ensure that microRNA quantitation is not affected by the variability that is potentially introduced at different experimental steps. U6 (RNU6A) and RNU6B are two commonly used endogenous controls in microRNA quantitation. The present study was designed to investigate inter-individual variability and gender differences in hepatic microRNA expression as well as to identify the best endogenous control/s that could be used for normalization of real-time expression data in liver samples.

**Methods:**

We used Taqman based real time PCR to quantitate hepatic expression levels of 22 microRNAs along with U6 and RNU6B in 50 human livers samples (25 M, 25 F). To identify the best endogenous controls for use in data analysis, we evaluated the amplified candidates for their stability (least variability) in expression using two commonly used software programs: Normfinder and GeNormplus,

**Results:**

Both Normfinder and GeNormplus identified U6 to be among the least stable of all the candidates analyzed, and RNU6B was also not among the top genes in stability. mir-152 and mir-23b were identified to be the two most stable candidates by both Normfinder and GeNormplus in our analysis, and were used as endogenous controls for normalization of hepatic miRNA levels.

**Conclusion:**

Measurements of microRNA stability indicate that U6 and RNU6B are not suitable for use as endogenous controls for normalizing microRNA relative quantitation data in hepatic tissue, and their use can led to possibly erroneous conclusions.

## Background

MicroRNAs are short (~22 bp) endogenous non-coding RNAs that regulate post transcriptional gene expression by binding to specific target mRNA transcripts and promoting their mRNA degradation/destabilization and/or translational inhibition [1]. They have been suggested to play important roles in defining normal tissue specific gene expression patterns [2]. Aberrant expression levels of microRNAs have been associated with several human diseases such as cancer [3,4], diabetes [5], metabolic disorders [6], neurological disorders [7] and cardiovascular diseases [8]. As microRNAs are relatively stable to degradation [9], they are being increasingly used as biomarkers for pathological states and clinical conditions [10,11].

An accurate determination of microRNA expression levels is, hence, key to the elucidation of their biology and roles in human diseases. Although several different strategies can be used for profiling microRNA expression levels, real time PCR or quantitative RT PCR (Q-RT PCR) is arguably the most used low to medium-throughput technique for measuring microRNA levels [12]. Given its high sensitivity and accuracy, real time PCR is frequently used to validate findings from genome-wide scans of microRNA expression. Results obtained using real time PCR are themselves, however, sensitive to experimental variation that can be introduced at several different steps, making the normalization strategy critical for obtaining accurate and reproducible results [13].

The most commonly used approach for normalization of real time PCR data is to consider one or more “invariant” single endogenous genes to control for the variability that is introduced at different stages of the real time PCR experiment, the key assumption, obviously being that these endogenous controls do not vary across the samples being studied [14]. Unfortunately however, to date, no standard endogenous controls have been identified for microRNA profiling and a large majority of microRNA expression profiling studies rely on the use of small nuclear RNAs such as U6 and/or RNU6B to control for experimental variability. There is limited evidence, however, to suggest that these controls do not vary across human tissue samples and as our study demonstrates, their use in quantitating hepatic microRNA levels can lead to incorrect interpretations and/or conclusions.

The goal of our study was to investigate the existence of inter-individual variability and gender differences in hepatic microRNA expression. We specifically focused on microRNAs that were (1) predicted (by Targetscan database) to have binding sites in 3’UTR regions of hepatic drug metabolizing enzymes and several key hepatic transcription factors known to be or potentially involved in regulating these drug metabolizing enzymes, and (2) known to be expressed in human liver based on literature. In addition we evaluated the use of mammalian U6 and RNU6B as endogenous controls, as both have been extensively used for normalizing hepatic microRNA expression levels. Our results demonstrate that both U6 and RNU6B are highly variable in expression and thus, not ideal for use as endogenous controls in normal human liver samples. Moreover we show that the use of U6 and RNU6B can lead to results that are highly different from those obtained when alternate stably expressed microRNAs are used as endogenous controls.

## Methods

### Study samples

50 human liver tissue samples (25 female, 25 male) were obtained from University of Minnesota’s Biological Materials Procurement Network facility (BIONET). The liver tissues used in this study were de-identified normal tissues obtained from human subjects that had been flash frozen and stored in liquid nitrogen. The research was carried out in compliance with the Helsinki Declaration and the use of the samples was reviewed and approved by University of Minnesota IRB.

### Isolation of total RNA

Total RNA was isolated from frozen human livers using the Mirvana miRNA isolation kit (Life Technologies, Grand Island, NY) followed by additional enrichment for RNA molecules of ~200 bp and less from the larger RNA species according to the manufacturer’s recommended protocol. Small RNA enrichment procedure allows more sensitive small RNA detection with less background as compared to the same assay used with total RNA. Briefly, approximately 35 mg of frozen liver tissue was homogenized in the lysis/binding buffer (Ambion) using TissueLyser LT (Qiagen) for 3 min at 50 Hz. Organic extraction followed by enrichment procedure for small RNAs was performed according to manufacturer’s protocol (Ambion). miRNA was eluted in 60 μl RNase-free water and stored at −80°C. The RNA quality, yield of each miRNA sample was obtained from A260 measurements using the NanoDrop 2000 (Thermo Fischer Scientific Inc). The RNA integrity number (RIN) was tested by using the Agilent 2100 Bioanalyzer (Agilent Technologies).

### microRNA reverse transcription

The isolated RNA was reverse transcribed using ABI miRNA reverse transcription kit (Part Number: 4366596) in combination with a multiplexed stem-loop primer pool which allowed us to simultaneously reverse transcribe 22 miRNAs and endogenous controls. The stem loop design ensured highly sensitive and specific amplification of mature microRNA targets [15]. The primer pool consisted of primers for 22 microRNAs, U6 and RNU6B –with the final concentration of each RT primer being 0.05 in a final volume of 1000 μL. The 22 microRNAs selected for this study were predicted (by Targetscan database) to have binding sites in 3’UTR regions of hepatic drug metabolizing enzymes and had indication of hepatic expression based on literature. Briefly 4.01 μl of total RNA was added to each RT reaction mix consisting of 6μls of the RT primer pool, 0.3 μl dNTPs with dTTP (100 mM), 3 μl of MultiScribe Reverse Transcriptase (50 U/μL), 1.5 μl of 10 RT Buffer, and 0.19 μls of RNase Inhibitor (20 U/μL) for a total reaction volume of 15 μl. Reverse transcription was performed at 16°C for 30 min, 42°C for 30 min followed by a final reverse transcriptase inactivation step at 85°C for 5 min.

### Real-time PCR amplification

We used custom designed 384 well TLDA (Taqman Low density Array) cards for real time PCR amplification of our microRNA targets and endogenous controls (U6 and RNU6B). The TLDA Cards were prepared as described in the TaqMan Array User Bulletin (Part no. 4371129). For each cDNA sample, 24 candidates were profiled in duplicate, allowing us to profile 8 samples per 384-well TLDA plate. As our RNA yields were >350 ng/4 μl we did not carry out pre-amplification. Briefly, 0.9 μl of RT product was added to 56.25 μL of TaqMan Universal Master Mix II, No AmpErase® UNG (2) (Part Number: 4324018) and 55.35 μL of Nuclease-free water for a total reaction mix of 112.50 μL. 100 μl of this was loaded onto each fill reservoir (corresponding to 2 lanes) of the TLDA plate. The TLDA plates were loaded onto an 7900HT system and default TLDA cycling conditions were used: 95°C for 10 min followed by 40 cycles of 95°C for 15 s and 60°C for 1 min. Following the reaction, the SDS 2.3 software was used to analyze the runs and these were transferred to an RQ study using Reference manager 1.2. The baselines and thresholds were set manually following visual examination of each run and raw Ct values were calculated. The RQ study output was analyzed using Data Assist v3.01 software from Applied Biosystems and Qbaseplus software from Biogazelle. Relative quantification was carried out using ddCT method using a common calibrator sample. Replicates/wells with Ct > 37 excluded.

### Stability analysis of candidate endogenous controls

We used two computational programs Normfinder [16,17], and GeNormplus [18] to compare and rank the candidate endogenous controls (U6 and RNU6B along with other microRNAs which could be used as candidate endogenous controls) on the basis of their stability (least variability in expression) in hepatic tissue. Along with U6 and RNU6B, the microRNAs analyzed as candidate endogenous controls included mir-152, mir-23b, mir-10a, mir-27a, mir-128a, mir-200a, mir-138 and mir-9. GeNormplus is integrated into Qbaseplus, which can be downloaded for a fully functional 15-day trial from Biogazelle (http://www.biogazelle.com/qbaseplus). Raw amplification (Ct) data from Reference Manager 1.2 was imported into Qbase plus for GeNormplus analysis. For stability and relative quantitation calculations, we used the option of calibrating the data to a specified sample. Apart from the stability calculations through the GeNormplus module, we also analyzed the relative quantitation data when it was normalized to (1) U6, (2) RNU6B and (3) geometric mean of mir-152 and mir-23b. The Mann Whitney test was used to test for gender differences between specific targets as a consequence of normalization.

Normfinder is a freely available as an excel add-in at the site: http://moma.dk/normfinder-software. As the Normfinder software can only process data that is on a linear scale, prior to analysis, we converted the Ct data into linear values by defining a specific sample as a calibrator and calculating the RQ values for other samples using the equation RQ = 2^^-(CtSample-CtCalibrator)^. For these calculations, the PCR efficiency was assumed to be 100%. As Taqman gene/microRNA expression assays are extensively validated for comparable PCR efficiency (which is reportedly close to 100%) and use the same amplification conditions, we felt that this was a reasonable assumption to make. In addition to ranking the genes based on their stability which is estimated using a linear mixed effects model, the Normfinder algorithm can also estimate both intra- and intergroup variance if groups are specified. We used gender to specify groups and analyzed variance within- and between male and female liver samples. As candidate microRNAs with a large inter-group (Male–female) variation can impact the stability calculations, we also re-evaluated the microRNAs for their stability after eliminating the candidates with large inter-group variation.

For both Qbaseplus and Normfinder calculations, we excluded replicates/wells with Ct > 37. A sample with missing data had to be excluded from the Normfinder analysis.

## Results

### MicroRNA amplification in human liver tissues and initial stability comparison using GeNormplus and Normfinder

**Figure 1:**
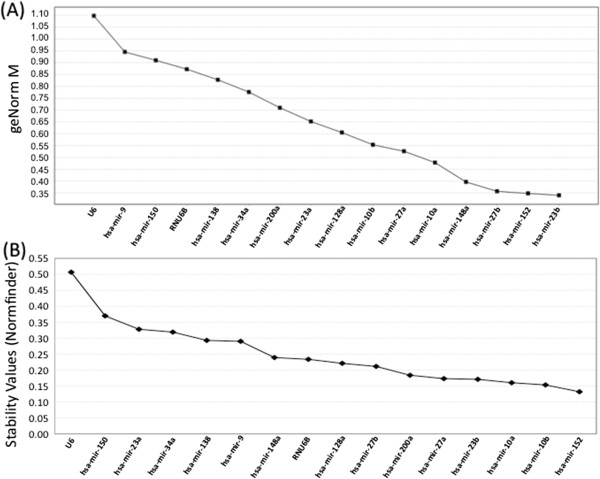
**Stability values for the candidate controls analyzed using (A) GeNormplus, and (B) NormFinder.** GeNormplus calculates the average expression stability value M for each candidate, which is calculated as the average pairwise variation between the candidate and all the other candidates. At each step the least stable candidate is excluded and M is recalculated until the most stable candidates are identified. Normfinder uses a model-based approach to calculate the stability value of each candidate based on its inter- and intra group variance.

We used a Custom TLDA card to amplify 22 microRNAs and the 2 endogenous controls, RNU6B and U6 (Table 1) in normal human liver tissues from 50 subjects (25 males and 25 females). All except 6 microRNAs (mir-613, mir-583, mir-137, mir-499, mir-206, mir-141) were amplified at appreciable levels. U6 (RNU6A) and RNU6B, two small nuclear RNAs that are frequently used to normalize microRNA data, were amplified as endogenous controls. To determine the suitability of U6 and RNU6B for use as reference controls in our analysis, we next compared their stability (a measure of variation in expression; high stability = low variation) with the stability of the microRNA targets amplified. We excluded microRNAs with missing data (mir-1 and mir-135a) from these stability calculations. Both GeNormplus and Normfinder identified U6 as the least stable (most variable) of all candidates analyzed (Figure 1A and Figure 1B respectively). RNU6B did not fare much better, being ranked 13^th^ of the 16 candidates analyzed for stability by GeNormplus (Figure 1A) and 9^th^ by Normfinder analysis (Figure 1B). mir-23b was identified as the most stable candidate in GeNormplus analysis while it was 4^th^ most stable in Normfinder rankings. Similarly mir-152 was the most stable in Normfinder rankings and 2^nd^ most stable in GeNormplus rankings.

**Table 1:** Details of microRNAs and endogenous controls profiled in this study

**ABI assay ID**	**Assay name**	**Mature microRNA sequence**	**Drug metabolizing genes and associated transcription factors targeted by microRNA (targetscan)**
460	hsa-miR-135a	UAUGGCUUUUUAUUCCUAUGUGA	CYP1B1, CYP4F2, NR3C1, NCOA1, HNF4γ, PPARα
1586	hsa-miR-613	AGGAAUGUUCCUUCUUUGCC	CYP2C8, CYP1A1, HNF4α, SLCO1B1
2216	hsa-miR-128a	UCACAGUGAACCGGUCUCUUU	CYP4F2, CYP2C9, CYP3A4, CYP3A5, CYP1A2, CYP2B6, HNF4γ, NCOA1, NR3C1, PPARα, PPARγ, PPARGC1α, RXRα
470	hsa-miR-148a	UCAGUGCACUACAGAACUUUGU	CYP2B6, NR1I2, HNF4α, RXRα, NCOA1, PPARα, PPARGC1α
399	hsa-miR-23a	AUCACAUUGCCAGGGAUUUCC	CYP2C18, CYP4F2, HNF4γ, NCOR1, NCOA1, NCOA6, PPARα, PPARGC1α
426	hsa-miR-34a	UGGCAGUGUCUUAGCUGGUUGU	NR1I2, HNF4a, NCOR2, PPARα, PPARγ, PPARδ, RXRα
408	hsa-miR-27a	UUCACAGUGGCUAAGUUCCGC	NCOA1, CYP1B1, CYP3A4, CYP4F2, HNF4γ, PPARα, RXRα, NR3C1
1129	mmu-miR-137	UUAUUGCUUAAGAAUACGCGUAG	CYP3A4
409	hsa-miR-27b	UUCACAGUGGCUAAGUUCUGC	PPARγ, HNF3γ, RXRα
2218	hsa-miR-10b	UACCCUGUAGAACCGAAUUUGUG	Ncor2, PPARα
463	hsa-miR-141	UAACACUGUCUGGUAAAGAUGG	CYP2C8, CYP2B6, CYP3A7, CYP3A4, CYP1B1, CYP2C18, NR3C1, PPARαa, PPARGC1α
400	hsa-miR-23b	AUCACAUUGCCAGGGAUUACC	NCOA6
473	hsa-miR-150	UCUCCCAACCCUUGUACCAGUG	CYP4A11, CYP3A4, CYP2A6, CYP1B1, CYP1A1, NR3C1, PPARα, PPARδ, PPARGC1α, HNF4α, NCOR1
475	hsa-miR-152	UCAGUGCAUGACAGAACUUGG	NR1I2
510	hsa-miR-206	UGGAAUGUAAGGAAGUGUGUGG	SLCO1B1
583	hsa-miR-9	UCUUUGGUUAUCUAGCUGUAUGA	CYP1B1, RXRα, NR3C1, PPARα, PPARδ, HNF4α, HNF4γ, NCOA1, NCOR2
2284	hsa-miR-138	AGCUGGUGUUGUGAAUCAGGCCG	CYP1A1, CYP1A2, RXRα, NR3C1, PPARα, PPARδ, NCOR1, NCOA1
1050	hsa-miR-506	UAAGGCACCCUUCUGAGUAGA	NR3C1, PPARα
502	hsa-miR-200a	UAACACUGUCUGGUAACGAUGU	YY1
387	hsa-miR-10a	UACCCUGUAGAUCCGAAUUUGUG	HNF4γ, NCOR1, NCOA6
2222	hsa-miR-1	UGGAAUGUAAAGAAGUAUGUAU	SLCO1B1
1973	U6 snRNA (NR_004394)	GTGCTCGCTTCGGCAGCACATATACTAAAATTGGAACGATACAGAGAAGATTAGCATGGCCCCTGCGCAAGGATGACACGCAAATTCGTGAAGCGTTCCATATTTT	
1093	RNU6B (NR_002752)	CGCAAGGATGACACGCAAATTCGTGAAGCGTTCCATATTTTT	

### Estimation of Intergroup variation (between genders)

**Figure 2:**
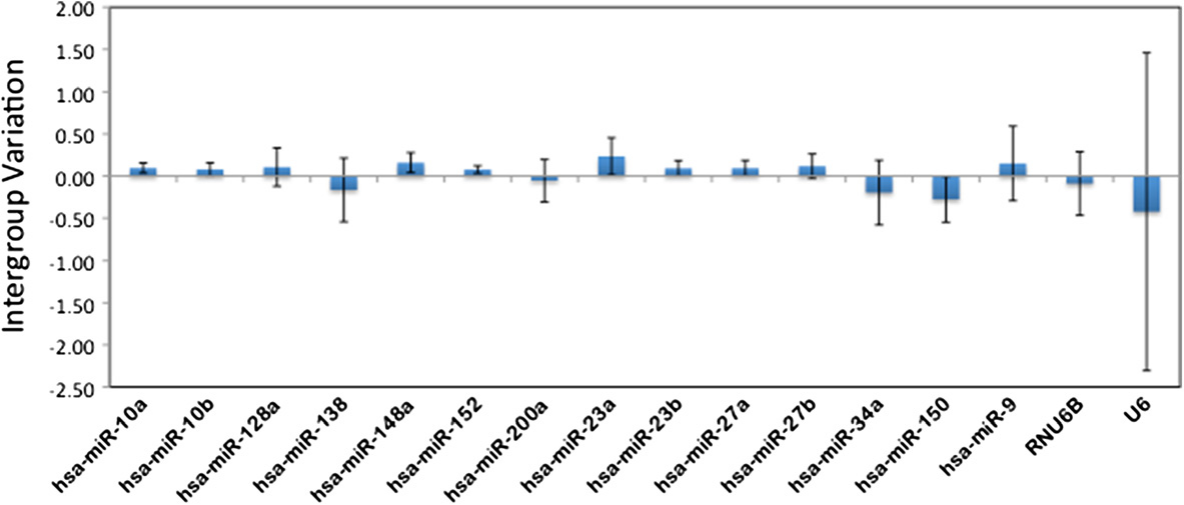
**Inter-group variation (between genders) in expression of candidate endogenous controls as estimated using Normfinder.** The error bars represent the average of the intragroup variation and provide a confidence interval for the intergroup variation for each candidate.

Eliminating candidates with large inter-group variation is expected to improve the estimates of variance and minimize the bias that any one target can have on outcome of stability calculations. For this analysis Normfinder was the program of choice as it allows the determination of both inter-group (if groups are specified) and intra-group variability. Normfinder uses a linear mixed effects model to calculate inter and intra-group variation. Using gender to specify groups, we determined which candidates had the highest inter-group variation between male and female liver samples (Figure 2). In this figure the error bars represent the average of the intragroup variation and are also helpful as they provide a confidence interval for the intergroup variation estimates. Of all the candidates analyzed, mammalian U6 had the highest inter-group and intra-group variability. This was very unexpected as U6 is frequently used as an endogenous control for microRNA expression analysis. U6 had by far the most skewed expression of all candidates analyzed, being expressed at higher levels in males compared to females. Several other candidates were expressed differentially between male and female livers including mir-150, mir-9, and mir-34a, these miRNAs particularly showing more than 2 fold variation. As the inclusion of such candidates with high inter-group variability can bias the stability calculations and the selection of reference genes, they were excluded and the remaining candidates reanalyzed for stability.

### Choosing a stable endogenous control for RQ analysis

To avoid any bias due to microRNAs that might be correlated in expression due to the fact that they belong to same family, we analyzed only one microRNA per family. This resulted in inclusion of mir-10a, mir-23b, mir-27b, and exclusion of mir-10b, mir-148a, mir-23a and mir-27a from further analysis.

**Figure 3:**
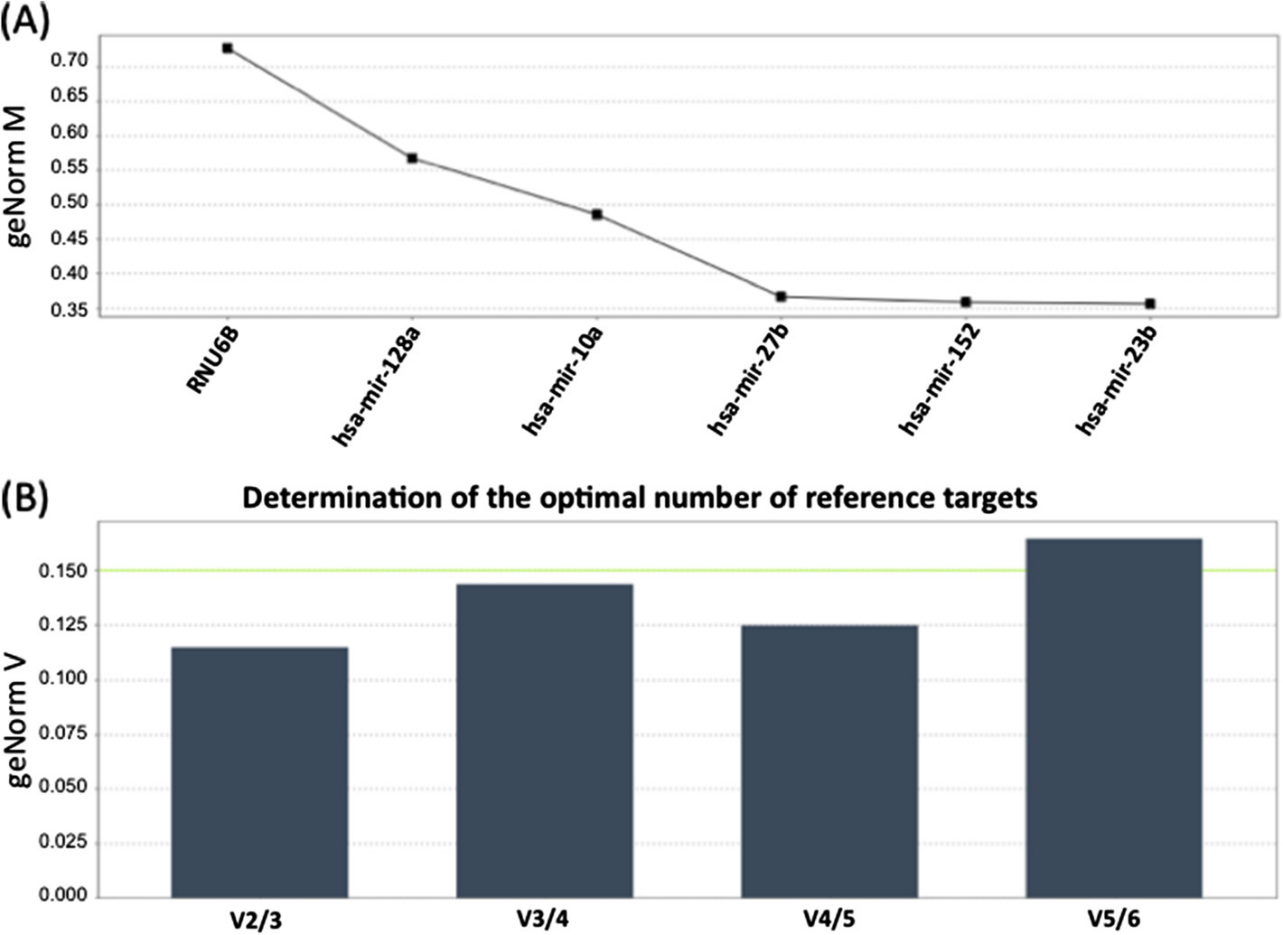
**GeNorm plus was used to determine (A) the most stable candidate/s as ranked based on M value, and (B) the optimal number of endogenous controls.** A normalization factor NF is calculated for at least 2 candidates starting from the most stable pair and the next most stable candidate/s are sequentially added to calculate additional NFs until the average pairwise variation between two sequential normalization factors (V = NF_n_/NF_n+1_, where n = number of controls used) falls below a set threshold (0.15).

An analysis of the best ranking candidates (RNU6B, mir-10a, mir-23b, mir-27b, mir-128a, and mir-152) using GeNormplus identified mir-23b as the most stable gene, followed by mir-152, mir-27b and mir-10a respectively (Figure 3A). Normfinder analyses also identified mir-152 and mir-23b as the two most stable candidates. Similarly both the programs agreed on RNU6B being the least stable among these candidates. An analysis using GeNormplus to identify the required number of endogenous controls for our analysis identified this number to be two, with a combination of mir-23b and mir-152 identified as the most stable of the reference candidates (Figure 3B).

### Impact of normalizer stability on relative quantification of microRNAs

As U6 and RNU6B were identified as being among the least stable of all the microRNAs/endogenous controls analyzed, we next wanted to study the potential impact of normalizing our data to U6 and to RNU6B versus normalizing it to stable microRNAs.

**Figure 4:**
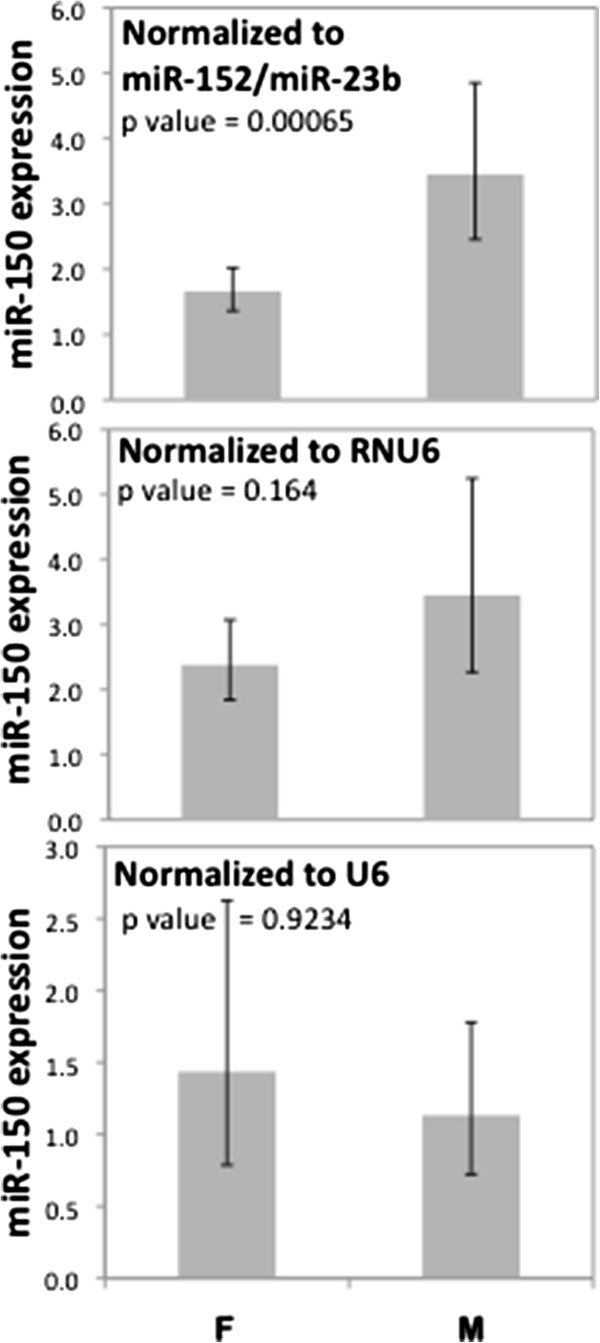
**Effect of Normalization on microRNA expression.** Gender differences (Males > Females) are observed in mir-150 expression when data is normalized to a combination of mir-152 and mir-23b (geometric mean) but this effect is lost when data is normalized to RNU6B or U6. Mann Whitney test was used to test for significance of gender differences between mir-150 expression as a consequence of normalization.

Figure 4 shows the effect of normalization on mir-150 levels (mean expression in males vs females) when data is normalized to either U6 or RNU6B or a combination of mir-152 and mir-23b, the two most stable microRNAs. Normalization to a combination of mir-152 and mir-23b identified females as having a significantly lower mean mir-150 expression as compared to males. Data normalization to RNU6B decreased the magnitude of difference, but still indicated females as having lower mir-150 levels than males. Normalizing the data to U6, however, did not show any difference among males and females with respect to the expression levels of mir-150. In addition to miR-150 which was the miRNA with most significant gender differences in expression, we observed similar associations for mir-138 and mir-34a where differences in expression levels between males and females were significant (p = 0.002 and p = 0.04, respectively) when data was normalized to a combination of mir-152 and mir-23b, but not when RNU6B or U6 were used for normalization. For mir-1, we observed significant gender differences by all three normalization methods (p < 0.05 for all).

To further study the impact of normalizer variability on relative quantitation of microRNA levels, we studied the correlations between mir-10b expression levels and other microRNAs when the data was normalized to U6, RNU6B or a combination of mir-152 and mir-23b.

As shown in Table 2, when the data is normalized to U6, mir-10b levels were highly correlated (r ≥ 0.8) with most of the targets analyzed. Normalization to RNU6B still showed mir-10b as having high correlations to several microRNAs, although the highest correlation was to mir-10a, a member of the same microRNA family. Normalizing the data to a combination of mir-152 and mir-23b however, identified only mir-10a as being highly associated with mir-10b while mir-27b was additionally identified as being negatively correlated to mir-10b.

**Table 2:** Correlations between mir-10b levels and other microRNAs when data is normalized to different endogenous controls

		**Normalized to U6**	**Normalized to RNU6B**	**Normalized to mir-152 and mir-23b**
target x	target y	r*	r*	r*
hsa-miR-10b	hsa-miR-10a	0.967	0.939	0.748
hsa-miR-10b	hsa-miR-128a	0.856	0.741	0.041
hsa-miR-10b	hsa-miR-138	0.834	0.59	0.231
hsa-miR-10b	hsa-miR-148a	0.922	0.824	0.205
hsa-miR-10b	hsa-miR-150	0.803	0.67	0.343
hsa-miR-10b	hsa-miR-23a	0.939	0.717	0.304
hsa-miR-10b	hsa-miR-27b	0.898	0.712	−0.452

*Pearson’s correlation coefficient.

We also looked at the correlation between RQ values for mir-10b when the data was normalized to U6, RNU6B or a combination of mir-152 and mir-23b. There was little or no correlation in mir-10b levels between U6 and RNU6B normalized data (r = 0.149) or between U6 and mir-152/mir-23b normalized data (r = −0.192), with some correlation being observed between RNU6B and mir-152/mir-23b normalized data (r = 0.644). These results indicate that U6 or RNU6B are not adequte for use as endogenous controls in liver tissue and that their use can lead to misleading results and incorrect data interpretation.

## Discussion

microRNAs constitute an integral part of cellular gene expression machinery and microRNA mediated regulation of gene expression represents a evolutionarily conserved paradigm that is essential for the proper functioning of each cellular/tissue type. Dysregulation of microRNA expression levels have been seen to be associated with a number of human diseases and pathological conditions [3-8]. The increasing use of microRNAs as biomarkers of human disease states has led to an exponential increase in microRNA expression profiling studies in recent years.

Although quantification of mature microRNAs presents a number of challenges such as the small size of microRNAs, sequence similarity between mature microRNAs and precursor microRNAs etc., real time PCR techniques (such as use of Taqman’s proprietary stem loop primers) allow us to amplify mature microRNAs with high sensitivity and specificity [15] and have thus become widely established in current use. Real time PCR results are, however, acutely dependent upon the normalization strategy followed for obtaining accurate and reproducible results [12,13]. A common practice in real time PCR data normalization has been the use of single endogenous controls that are supposedly “invariant” in expression across normal and/or diseased samples [14]. MicroRNA quantitation, however, so far suffers from a lack of reference controls that have been standardized for use in specific tissue types and several studies have utilized small RNAs such as U6 or RNU6B as endogenous controls [19].

With a goal of studying inter-individual variation and gender differences in hepatic microRNA expression, we carried out a microRNA expression profiling study in 50 human liver samples, with 25 male and 25 female samples. The candidates chosen for the study included microRNAs with predicted binding sites in a set of hepatic drug metabolizing genes and associated transcription factors. Mammalian U6 and RNU6B were included as the potential endogenous controls.

To identify the best endogenous controls for use in data analysis, we next evaluated the amplified candidates for their stability (least variability) in expression using Normfinder and GeNormplus, two commonly used programs [20-22]. Normfinder uses a linear mixed effects model for estimating the amplification variability, and if groups are specified, provides estimates of inter-group as well as intra-group variability for each candidate. GeNormplus on the other hand, analyzes each candidate for its pairwise variation with all other candidates being analyzed. The candidate with the highest average pairwise variation is eliminated sequentially and the data recalculated for remaining candidates at each step. A reiteration of this process is used to identify the genes with the most stable expression. To avoid any bias due to genes expressed differentially between males and females, we excluded the genes identified by Normfinder to be having large male–female variability. We also excluded microRNAs with missing data and included only one microRNA per family. This is especially important as GeNormplus calculates stability based on pairwise variation and thus GeNormplus analysis may be susceptible to artifacts when genes, which are co-regulated (and hence, correlated in expression), are analyzed [16].

Both Normfinder and GeNormplus identified U6 to be among the least stable of all the candidates analyzed. Based upon Normfinder analysis, U6 was in fact identified as the candidate with the most inter-group variation (difference in expression between males and females; U6 was more highly expressed in males compared to females) and also the candidate with the most intra-group variation (variation within male and female groups). Normalizing the data to U6 would thus have indicated all other candidates as having higher levels in females than in males.

The GeNormplus analysis of all male and female livers combined also identified U6 to be the least stable of all candidates analyzed. RNU6B, though not as variable as U6, was also identified as being highly variable by both programs.

Both Normfinder and GeNormplus identified mir-152 and mir-23b as the two most stable candidates. An analysis using GeNormplus to identify the minimum number of endogenous controls required, identified this number as two, with the combination of mir-23b and mir-152 being the most stable. As mir-23b is much more abundant than mir-152, it makes sense to take a geometric mean of both for data normalization.

As the use of variable controls such as U6 in data normalization is likely to introduce added variability in real time PCR results, we analyzed our data for the potential impact of normalizing to U6 or RNU6B, and compared the RQ results obtained to when the data was normalized to a combination of mir-152 and mir-23b, (the most stable pair identified by both Normfinder and GeNormplus). Significant gender differences (females < males) were observed in mir-150 expression when data was normalized to mir-152 and mir-23b, but when not data was normalized to U6. There was also little to no correlation in the RQ results obtained when data was normalized to U6 and when it was normalized to a combination of mir-152 and mir-23b.

Normalizing to U6 (and RNU6B also) showed mir-10b as having very high correlations (r > 0.8) to all the other microRNAs tested, which is highly improbable. However, normalization to a combination of mir-152 and mir-23b allowed us to discriminate more accurately between the microRNA relationships and identified mir-10a as the only microRNA with high correlation to mir-10b expression. This correlation is both plausible and likely given that both mir-10a and mir-10b belong to the same microRNA family and microRNAs from the same family are significantly more likely to be co-expressed [23]. At the same time, normalizing to a combination of mir-152 and mir-23b also allowed us to identify a negative relationship between mir-10b and mir-27b. Although we do not know the biological relevance of this association, especially in context to hepatic compartment, inverse relationships of mir-10b and mir-27b have been previously observed in other studies too [24,25]. For example using a steatotic L02 cell model, Zheng et al. [24] demonstrated that mir-10b was up-regulated, and mir-27b was down-regulated in non-alcoholic fatty liver disease (NAFLD). Additionally, up regulation of miR-10b and down-regulation of miR-27b has been observed in oesophageal cancer [26].

Our results suggest that U6 which has been frequently used as an endogenous control, is not an appropriate control when evaluating expression profiles of miRNAs in liver. Although we identified mir-152 and mir-23b miRNAs as most stable and appropriate endogenous controls for normalizing expression of hepatic miRNAs involved in regulation of drug metabolizing genes, the relevance and applicability of these findings to other disease conditions/tissue types should be systematically evaluated before making a decision on the choice of appropriate endogenous controls. As a further expansion of this work, we are planning to evaluate the utility of miR-152 and mir-23b as endogenous controls in hepatic carcinoma samples.

U6 and RNU6B are commonly used as normalizing genes in microRNA quantitation studies. Despite limited evidence to suggest that they indeed demonstrate invariant expression across normal tissues or disease samples, U6 (as also RNU6B) continue to be used in large numbers. A search for 2012 papers on Google scholar for “normalized to U6” AND (hepatic OR liver) terms identified 163 papers. We are not aware of any study that has tested the suitability of U6 and/or RNU6B as endogenous controls in human liver tissues. It is important to publish our findings as a large number of pharmacogenomic studies focusing on microRNA mediated regulation in the hepatic compartment continue to use U6 and RNU6B as endogenous controls.

Finally, an additional challenge presented by microRNA quantitation is the relatively low amounts of the microRNAs present. microRNAs are suggested to constitute around 0.01-0.1% of the total RNA present and the use of highly expressed endogenous controls such as U6 will likely not follow similar amplification kinetics as that of low abundance microRNAs. Hence, normalizing the data to microRNAs instead of small RNAs can lead to better results.

## Conclusion

In conclusion we recommend that prior to their use as endogenous controls, any candidate/s should be tested for their stability. There are a number of freely available programs that allow the assessment of reference genes for stability. The use of U6 or RNU6B should be discouraged for hepatic expression profiling studies as their use can lead to incorrect conclusions/interpretations.

## Abbreviations

RQ: Relative quantitation; TLDA: Taqman low density array; miR: microRNA.

## Competing interests

Authors have no competing interests to disclose.

## Authors’ contributions

VL designed and carried out the study, helped in acquisition of samples, performed analysis and wrote the manuscript; YG-P, performed miRNA and RNA isolations, participated in study design and helped in writing the manuscript; WG, helped in data analysis, participated in study design and contributed in manuscript writing; JL, helped in analysis, acquisition of samples, participated in study design and manuscript writing. All authors read and approved the final manuscript.
